# Semipilot Scale Synthesis of Zeolite A from Kaolin Filler: Process Optimization with Boiler Steam, Mathematical Modeling, and Cu^2+^ Adsorption

**DOI:** 10.1021/acsomega.5c13534

**Published:** 2026-05-28

**Authors:** Emerson Cardoso Rodrigues, Camila Santana Dias, Josiel Lobato Ferreira, Bruno Maués Farias, Diego Cardoso Estumano, Bruno Marques Viegas, José Antônio da Silva Souza, Emanuel Negrão Macêdo

**Affiliations:** † Engineering of Natural Resources of the Amazon, 37871 Federal University of Pará, Belém, PA 66075-110, Brazil; ‡ Faculty of Chemical Engineering, Federal University of Pará, Belém, PA 66075-110, Brazil; § Faculty of Biotechnology, Federal University of Pará, Belém, PA 66075-110, Brazil; ∥ Simulation and Computational Biology Laboratory, High Performance Computing Center, Federal University of Pará, Belém, PA 66075-110, Brazil; ⊥ Graduate Program in Chemical Engineering, Federal University of Pará, Belém, PA 66075-110, Brazil

## Abstract

This work reports the semipilot-scale synthesis of zeolite A using a boiler as an unconventional thermal energy source and Amazonian kaolin filler as the Si/Al source. By enabling more effective heat transfer and reducing reaction times without compromising product quality, the proposed method enhances conventional synthesis routes. Zeolite A formation was confirmed by structural characterization using XRD, XRF, TG/DTA, and SEM. Adsorption experiments of Cu^2+^ ions were conducted at 25, 50, and 75 °C, showing maximum adsorption capacities of 107.5, 124.1, and 119.9 mg g^–1^, respectively, demonstrating high metal removal performance. Langmuir, Freundlich, and Sips isotherms were used to model the adsorption equilibrium, and parameters were estimated via a Markov chain Monte Carlo method. The Langmuir isotherm provided the best fit at 25 °C, whereas the Sips isotherm showed superior performance at 50 and 75 °C, as indicated by both the coefficient of determination and the Bayesian Information Criterion. The results highlight the potential of this route as a scalable and efficient alternative for zeolite A synthesis, capable of producing high-quality material in a reduced synthesis time and demonstrating effective performance in environmental applications.

## Graphical abstract

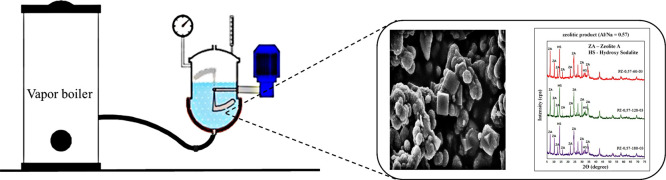

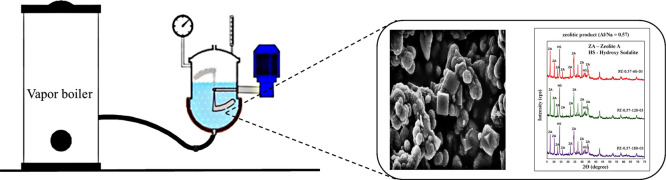

## 1 Introduction

The mining sector is one of the main pillars of the Brazilian economy, attracting significant investments. In 2023, investment in this sector exceeded 30 billion Brazilian reais (BRL). In the same year, investments in mineral research reached approximately 2.3 billion BRL. This development is reinforced by the mineral sector’s greater contribution to the Brazilian economy, where total production exceeded 0.26 trillion BRL, and by its significant impact on the labor market, with the creation of approximately 846,600 jobs in the first half of 2022.^1^

Regarding kaolin, total production of this mineral in Brazil in 2023 reached around 0.85 billion BRL, with income from the processed product amounting to 811.40 million BRL. The state of Pará stands out as the main producer of this consumer good in the period, with 602,980.43 tons of processed kaolin, compared to more than 800,000 tons produced in the country, generating a total income in the state of about 480 million BRL.^1^

The physical and chemical properties and low cost of kaolin enable its use in various industrial segments, such as paints, cosmetics, plastics, and rubber. However, its greatest use is in the paper industry, where it acts as a coating and filling agent, giving the paper surface uniform density and porosity, which are essential for obtaining high-quality prints.^2–4^

In addition, due to its resistance to pH variations, thermal and hydrothermal stability, molecular selectivity, and presence of exchangeable cations, kaolin has gained prominence as a raw material for the synthesis of zeolites.^5^

Zeolites are highly ordered crystalline aluminosilicates widely used as molecular sieves, catalysts, and adsorbents, as well as in cracking, separation, and purification processes. Among them, zeolite A stands out for its commercial use, mainly in water decontamination through the adsorption of metal ions, an application that has shown promising results.^6–10^

Even though the hydrothermal method is widely used to synthesize zeolite A, the advances documented in the literature are still mostly restricted to laboratory-scale settings and heavily rely on lengthy crystallization times and traditional heating configurations.^11–16^ There are currently few studies that systematically address the use of alternative thermal sources, process adaptation to larger scales, and synthesis time reduction. Furthermore, not much research examines how these changes affect the process’s operational viability and the final product’s functional performance. Thus, there is a glaring need to develop more effective and scalable synthesis routes to produce zeolite A. The central objective of this study is to evaluate the efficient production of zeolite A at an enlarged scale using filler kaolin via an alternative synthesis route that employs a boiler as the heat source. Unlike conventional laboratory-scale methods, which are often limited by long reaction times and scale-up challenges, the proposed approach significantly reduces synthesis time while maintaining material quality. The synthesis was carried out at a semipilot scale, using a larger-volume closed reactor under controlled temperature and pressure. This demonstrates the practical potential of the method for scale-up and future implementation in more realistic production scenarios.

The use of a boiler as a thermal source represents an advancement over traditional routes by providing efficient heating compatible with larger scales, thereby improving the operational feasibility of the process. Furthermore, the application of the synthesized zeolite A for Cu^2+^ ion adsorption, combined with Bayesian inference via the Markov chain Monte Carlo (MCMC) method to estimate isotherm parameters, integrates synthesis, environmental application, and advanced statistical analysis within a single study, while also allowing quantification of parameter uncertainty, capturing correlations between parameters, and providing a probabilistic framework for model predictions.

In this context, the Cu^2+^ ion was selected as a model contaminant due to its recognized environmental relevance, its association with mining and mineral processing activities, and its widespread use as a reference ion in adsorption studies. This integrated approach reinforces the relevance of the proposed process, particularly in the Amazon region, where mining activities intensify the presence of heavy metals in the environment, and highlights the potential of the developed route as an alternative to conventional zeolite A synthesis methodologies.

## 2 Materials and Methods

### 2.1 Starting Material and Reagents

The kaolin filler used as a source of silica and alumina in this investigation comes from the classification stage, which is part of the kaolin beneficiation process,^2^ carried out in the city of Barcarena, in the state of Pará, Brazil. Sodium hydroxide (NaOH) was used in the synthesis of the zeolitic material, while copper sulfate pentahydrate (CuSO_4_.5 H_2_O) and sulfuric acid (H_2_SO_4_) were used in the adsorption experiments.

A 5.0 M sodium hydroxide solution was prepared by dissolving analytical-grade NaOH (Casa da Química Industrial e Comércio Ltd.a., Brazil) in distilled water. Similarly, a 1000 ppm copper solution was prepared from analytical-grade copper sulfate pentahydrate (CuSO_4_·5H_2_O, Casa da Química Industrial e Comércio Ltd.a., Brazil) using distilled water, while a 1.0 M sulfuric acid solution was obtained by diluting analytical-grade H_2_SO_4_ (65–98 wt %, density 1.84 g·cm^–3^, Casa da Química Industrial e Comércio Ltd.a., Brazil) with distilled water.

### 2.2 Synthesis of Zeolite Material

Initially, the kaolin filler was calcined at 600 °C for 2 h to remove adsorbed water and impurities, ensuring maximum efficiency in the subsequent adsorption experiments.^17^ The synthesis method used to produce the zeolitic product was hydrothermal on a semipilot scale.^41,51^ The material obtained after calcination was introduced into a stainless-steel reactor with an internal volume of approximately 0.018 m^3^. A total mass of 4.5 kg of calcined kaolin was charged into the reactor and mixed with a 5 M NaOH solution to adjust the mixture to an Al/Na ratio of 0.57. The hydrothermal synthesis was carried out at a temperature ranging from 110 to 120 °C under a pressure of 1.5 kgf·cm^–2^. The zeolite synthesis process was maintained for 3 h.

The heating system was supplied by steam generated from a boiler (model: VMV 1P 150), with a capacity of 150 kg·h^–1^, a heating surface area of 6.3 m^2^, and a diesel oil consumption rate of 11 kg·h^–1^. After the synthesis period, the reactor was cooled to room temperature, and the suspension was filtered to separate the phases until neutral pH was reached. Following the filtration step, the zeolites were dried in an oven for 24 h at 110 °C.

During the synthesis process, aliquots were withdrawn at 30 min intervals to investigate the kinetics of zeolite formation on a semipilot scale. Each collected sample was immediately cooled to room temperature to quench the reaction, followed by filtration to separate the solid phase. The recovered solids were washed with distilled water until neutral pH was reached and subsequently dried prior to further characterization. Figure 1 shows the equipment used for zeolite synthesis.

**Figure 1:**
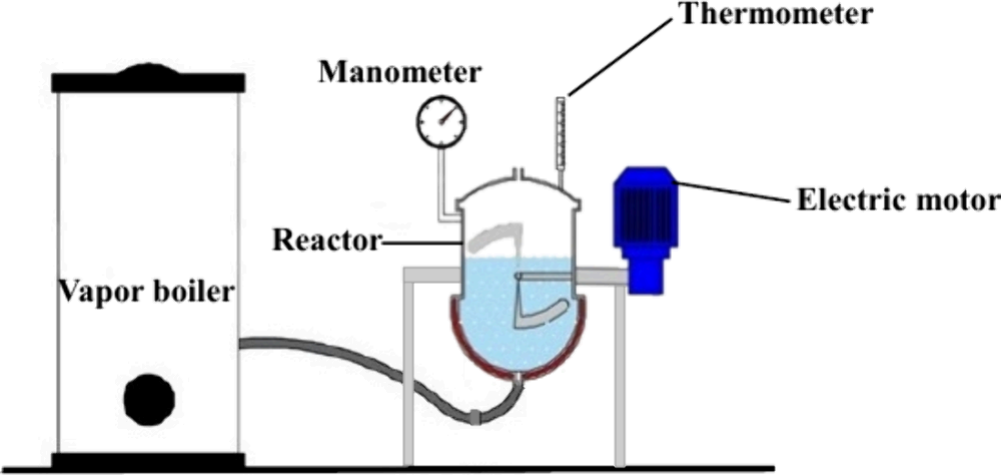
Schematic representation of the reactor system employed for zeolite synthesis.

### 2.3 Adsorption Tests

The zeolite produced was subjected to a calcination process at a temperature of 400 °C before the adsorption tests were carried out.^18^ Batch adsorption tests were performed with a copper sulfate solution at a concentration of 1000 ppm. A 1 M H_2_SO_4_ solution was used to adjust the pH.

The concentration of copper in the solutions was determined by UV–vis spectrometry at 800 nm. This technique was selected due to its sensitivity for Cu^2+^ detection and its lack of interference from the sulfate anion present in the aqueous medium. Measurements were performed following standard procedures, ensuring reliable quantification of copper for the adsorption experiments. The calibration curve used for Cu^2+^ quantification is provided in the Supporting Information. An experimental uncertainty of 1% (expressed as standard deviation) was considered based on the analytical precision of the concentration measurements.

#### 2.3.1 Adsorption Kinetics

It was used 0.02 g of the synthesized product and added to Erlenmeyer flasks containing 100 mL of a solution with copper ions Cu^2+^, whose concentrations of this adsorbate were evaluated in each test: 80, 200, 300, 500, and 1000 ppm. The temperature adopted was 25 °C with agitation at 150 cycles/min for 240 min.

#### 2.3.2 Adsorption Equilibrium

The adsorption equilibrium tests were conducted at temperatures of 25, 50, and 75 °C. For each temperature evaluated, six solutions with different Cu^2+^ ion concentrations were used (20, 60, 100, 200, 300, 500, and 1000 ppm). These concentrations were selected to represent untreated or highly concentrated industrial effluents and to enable a reliable evaluation of adsorption capacity and equilibrium modeling. The total experiment time was 240 min, with a stirring rate of 150 cycles/min, maintaining the same conditions adopted in the kinetic tests.

Table 1 shows the Langmuir, Freundlich, and Sips isotherms^19–26^ used to verify which would be most suitable for the equilibrium data obtained.

**Table 1:** Most Relevant Parameters and Assumptions of the Isotherms Used

Isotherm	Model	Hypotheses	Parameters
Langmuir	$q_{e}=\frac{q_{m}KC_{e}}{\left(1+KC_{e}\right)}$	Monolayer, uniform surface	*q*_*m*_ and *K*
Freundlich	$q_{e}=K_{F}C_{e}^{1/n}$	Multilayered, heterogeneous surface	*K*_*F*_ and *n*
Sips	$q_{e}=\frac{K_{S}C_{e}^{\beta_{S}}}{1+a_{S}C_{e}^{\beta_{S}}}$	The sites are not energetically homogeneous.	*a*_*S*_, *K*_*S*_ and *β*_*S*_

In these equations, *q*_*e*_ (mg/g) the amount adsorbed at equilibrium, *q*_*m*_ (mg/g) the maximum adsorption capacity, *K* (L/mg) the Langmuir constant, *C*_*e*_ (mg/L) the concentration of the solution at equilibrium, *K*_*F*_ (L/mg) and *n* (−) are parameters of the Freundlich isotherm. *K*_*S*_ (L/mg) is the sips constant, *a*_*S*_ (L/mg) is the affinity coefficient of this isotherm and *β*_*S*_ (−) is the heterogeneity coefficient.

### 2.4 Estimation of Isothermal Parameters

The isotherm parameters adopted in this study were estimated using the Markov Chain Monte Carlo method within a Bayesian framework. This method is based on the random generation of samples from the posterior probability distribution within the parametric space until an equilibrium distribution is reached. This equilibrium indicates the region of highest probability for the estimated parameter values, ensuring that they are statistically adequate to represent the experimental profile with the adopted model.^27–39^

After the parameter estimation process, the quality of the fit between the isotherms and the experimental data was evaluated using the coefficient of determination (R^2^) and the Bayesian Information Criterion (BIC), eq 1.

1 $$\mathrm{BIC}=-2\log\left[p\left(Y|P\right)\right]+N_{p}\log\left(N_{\mathrm{med}}\right)$$

where *N*_p_ represents the number of parameters to be estimated, *N*_med_ the number of measurements used.

### 2.5 X-ray Diffraction

The X-ray diffractometer used was the X’PERT PRO MPD (PW 3040/60) model from PAnalytical, with a PW3050/60 goniometer (Theta/Theta) and a ceramic X-ray tube with a Cu anode (Kα1 = 1.540598 Å), model PW3373/00, with a long focus, 2200W, 60kV. A Ni Kβ filter was used. The detector used was the X’Celerator, RTMS (Real Time Multiple Scanning) type, operating in Scanning mode with an active length of 2.122°. The following instrumental conditions were used: scan from 5° to 75° 2θ, voltage: 40 kV, current: 30 mA, step size: 0.02° 2θ and time/step: 5s, 1/2° divergent slit and 1° antiscattering; 10 mm mask, sample movement: Spinning, with 1 rotation/s.

The data was obtained using X Pert Data Collector software, version 2.1a, and processed using X Pert High Score software, version 2.1b, from PAnalytical, consulting the PDF database (Powder Diffraction File) of the ICDD (International Center for Diffraction Data).

### 2.6 X-ray Fluorescence Spectrometry

The spectrometer used to determine the chemical composition of kaolin was the sequential WDS, model Axios Minerals from PAnalytical, with a ceramic X-ray tube, rhodium (Rh) anode, and maximum power level of 2.4 KW. The preparation method was the fused disc method: 1 g of sample +6 g of flux (lithium tetraborate - Li_2_B_4_O_7_), mixed and melted at 1000 °C for 10 min. Data acquisition and processing were performed using the SuperQ Manager software from PAnalytical.

### 2.7 Differential Thermal Analysis and Thermogravimetric Analysis

The thermal decomposition curves of the kaolin filler were obtained using a simultaneous thermal analyzer (STA 1000/1500, PL Thermal Sciences, Stanton Redcroft Ltd.) equipped with a vertical cylindrical furnace and a digital converter coupled to a microcomputer. A platinum (87%)/rhodium (13%) thermocouple and an alumina crucible were used for samples of approximately 10 mg. The analyses were carried out under an inert atmosphere (nitrogen flow rate) at a heating rate of 20 °C/min, over the temperature range from 25 to 1000 °C.

### 2.8 Granulometric Analysis

The equipment used for this analysis was the Laser Particle Sizer analysette 22 from Fritsch GmbH, with the aid of MaScontrol software, also from Fritsch GmbH, for data acquisition and generation of the particle size distribution graph for the kaolin filler and metakaolin.

### 2.9 Scanning Electron Microscopy

Scanning electron microscopy analysis of kaolin and metakaolin was performed using Zeiss equipment (model LEO 1430). For the synthesized zeolitic product, the samples were metallized with gold using an EMITECH K550 metallizer. Metallization was performed by the interaction between a pure Au target and Ar ions (argon gas) at a pressure of 2.10^–1^ mbar and a current of 25 mA for 150 s, resulting in the deposition of a film with an average thickness of ± 15 nm on the samples. The samples were structured on aluminum supports with a diameter of 10 mm using carbon adhesive tape.

## 3 Results and Discussion

The results obtained show the composition of the starting material using the X-ray Fluorescence (XRF) technique. Next, the thermal behavior of this material is analyzed using Thermogravimetry and Thermogravimetric Derivative analyses, allowing an analysis of the changes that occur during heating. The granulometric analysis shows the behavior of both kaolin and metakaolin, providing information on the particle size distribution in each material. Finally, the results obtained by X-ray Diffraction (XRD) and Scanning Electron Microscopy (SEM) are presented, addressing the results for kaolin, metakaolin, and the synthesized zeolite.

### 3.1 X-ray Fluorescence of Kaolin Filler

Table 2 shows the chemical composition of the kaolin filler analyzed by X-ray fluorescence in comparison with results from the literature obtained by Zhou et al.^40^

**Table 2:** Chemical Composition of Kaolin Filler by X-ray Fluorescence

Oxides	This work	Zhou et al.^40^	Mahima Kumar et al.^41^	Abubakar et al.^42^
Al_2_O_3_	37.32	39.95	36.27	42.90
Fe_2_O_3_	0.53	–	0.347	0.31
Na_2_O	0.19	–		–
P_2_O_5_	0.10	–		0.01
SiO_2_	47.08	46.51	41.71	55.40
CaO	–	–	0.451	0.07
MgO	–	–	0.428	0.04
K_2_O	–	–	–	1.30
MnO				0.01
TiO_2_	0.42	–	1.51	0.05
Loss on Ignition	14.36	13.95	13.16	–

The results presented in Table 2 reveal that the Al_2_O_3_ (37.32%) and SiO_2_ (47.08%) contents predominantly characterize the clay mineral kaolinite as the main component of the material investigated in this study. This composition suggests an adequate Si/Al ratio for the formation of zeolites.^43,44^ Although the composition of materials of this nature can be influenced by geographical factors, it is worth noting that the values obtained in this study are like others reported in the literature.^40–42^ This study also highlighted the presence of a low percentage of impurities, the main ones being 0.53% Fe_2_O_3_, 0.42% TiO_2_, and 0.19% Na_2_O, corroborating previous findings.

### 3.2 Differential Thermal Analysis and Thermogravimetric Analysis

Figure 2 shows the differential thermal analysis (DTA) and thermogravimetric analysis (TG) curves of the kaolin filler. The TG curve shows a total mass loss of approximately 14%. In the DTA curve, a distinct peak at 536 °C corresponds to an endothermic process related to the formation of metakaolin, resulting from the dehydroxylation of kaolinite and the generation of an amorphous structure.^45^ A second exothermic peak at 992 °C indicates the crystallization of mullite or aluminum–silicon spinel.^2,46^

**Figure 2:**
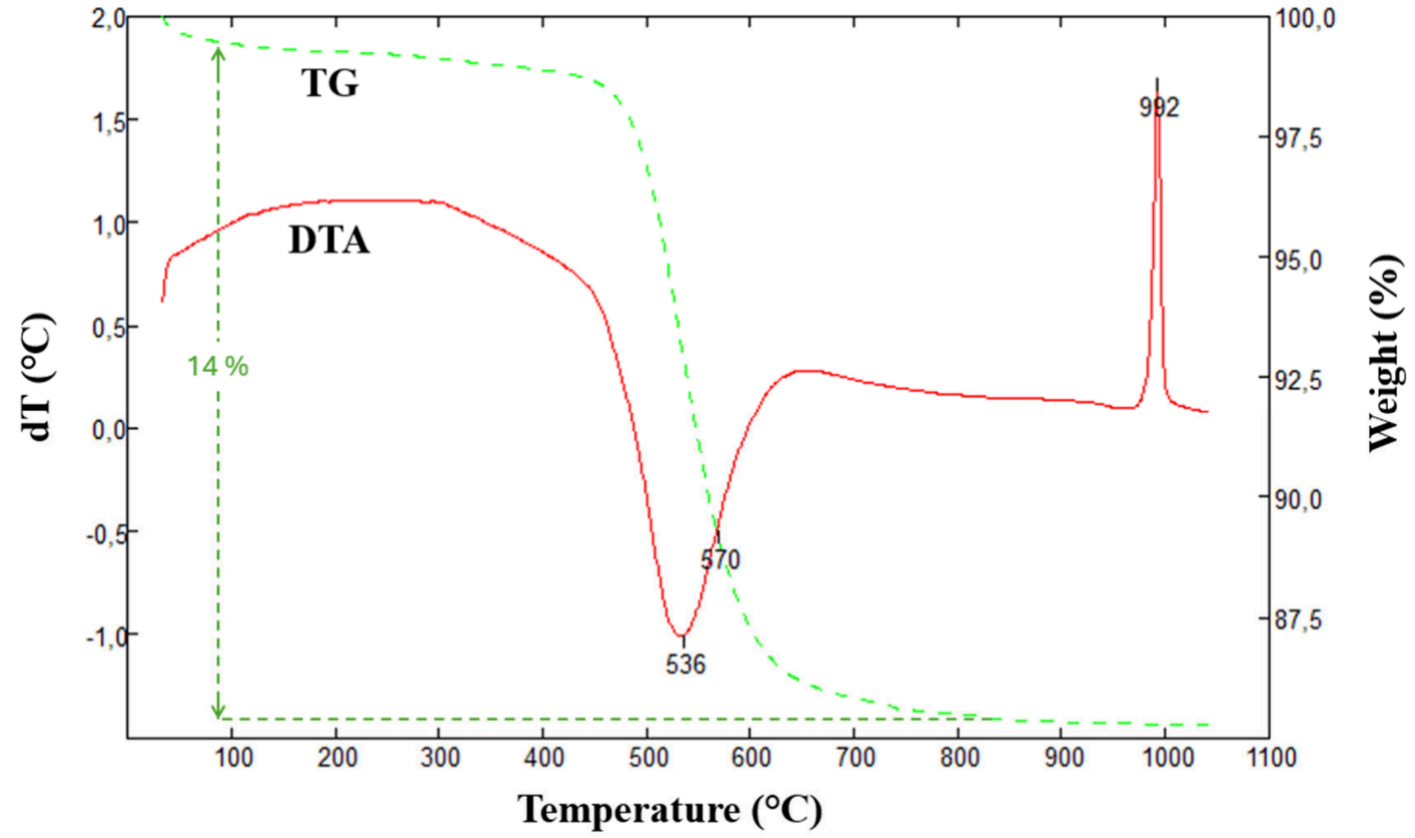
TG and DTA curves of the kaolin filler.

### 3.3 Granulometric Analysis

According to Santos,^47^ coating kaolins are characterized by having 75–95% of their particles with a diameter below 2 μm, whereas filling kaolins have 20–65% of their particles below this size. The higher proportion of fine particles in coating kaolins provides better surface coverage and smoothness in ceramic applications, while filler kaolins, with a larger fraction of coarser particles, are typically used to reduce costs, adjust rheological properties, and influence the porosity and reactivity of the final material.

Figure 3 shows the results of the kaolin granulometric analysis, indicating that approximately 30% of the particles have a diameter smaller than 2 μm, classifying this material as a filler kaolin. This particle size distribution contributes to reducing zeolite synthesis costs compared to coating kaolins.^48^

**Figure 3:**
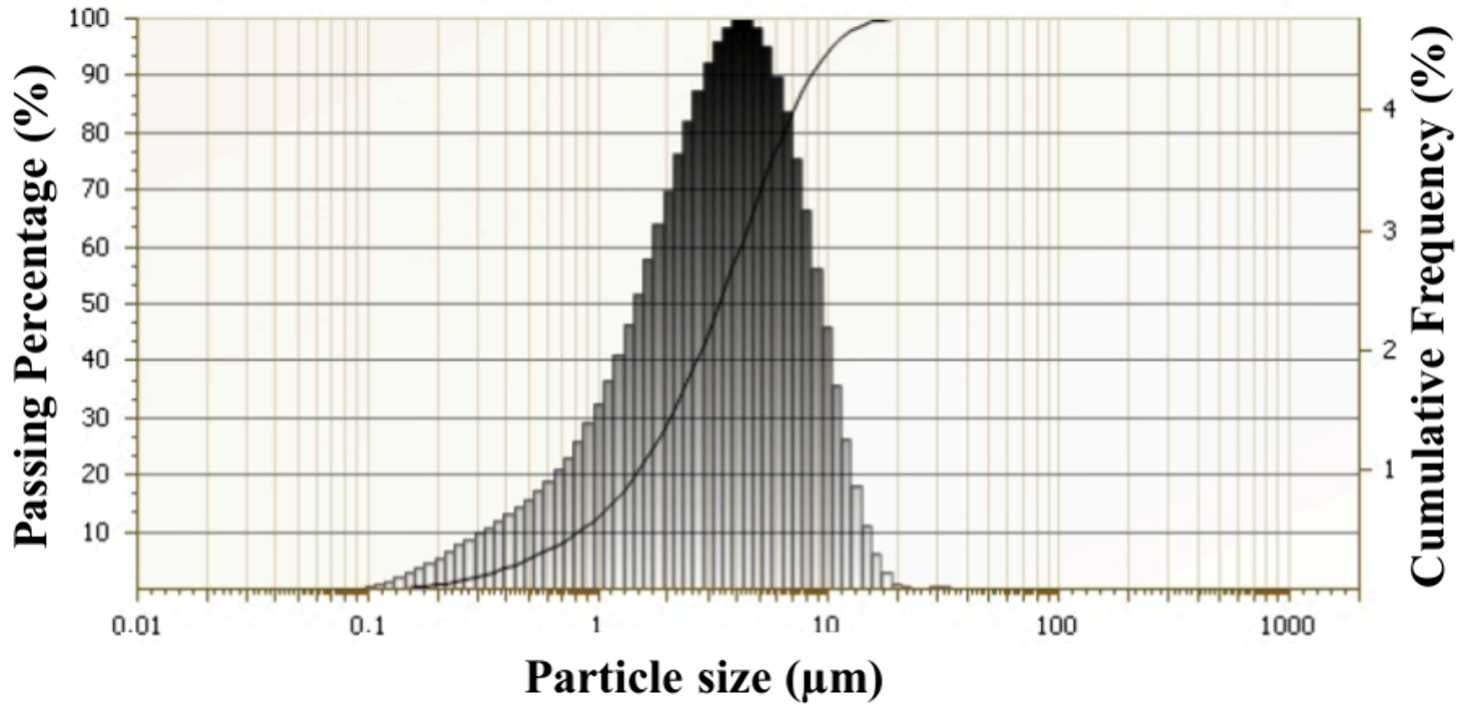
Particle size distribution of kaolin.

Figure 4 shows a decrease in the number of particles in the 0.1–1 μm range, which can be attributed to the calcination of kaolin. During the transformation of kaolinite to metakaolinite, smaller particles tend to agglomerate due to sintering. This is reflected in the particle size distribution: for kaolin (Figure 3), d10 = 0.8 μm, d50 = 3.4 μm, and d90 = 8.5 μm, whereas for metakaolin (Figure 4), d10 = 1.6 μm, d50 = 4.7 μm, and d90 = 10.5 μm, indicating a clear agglomeration of particles. These results demonstrate that the calcination step effectively modifies the granulometry, which is important for the subsequent zeolite synthesis.

**Figure 4:**
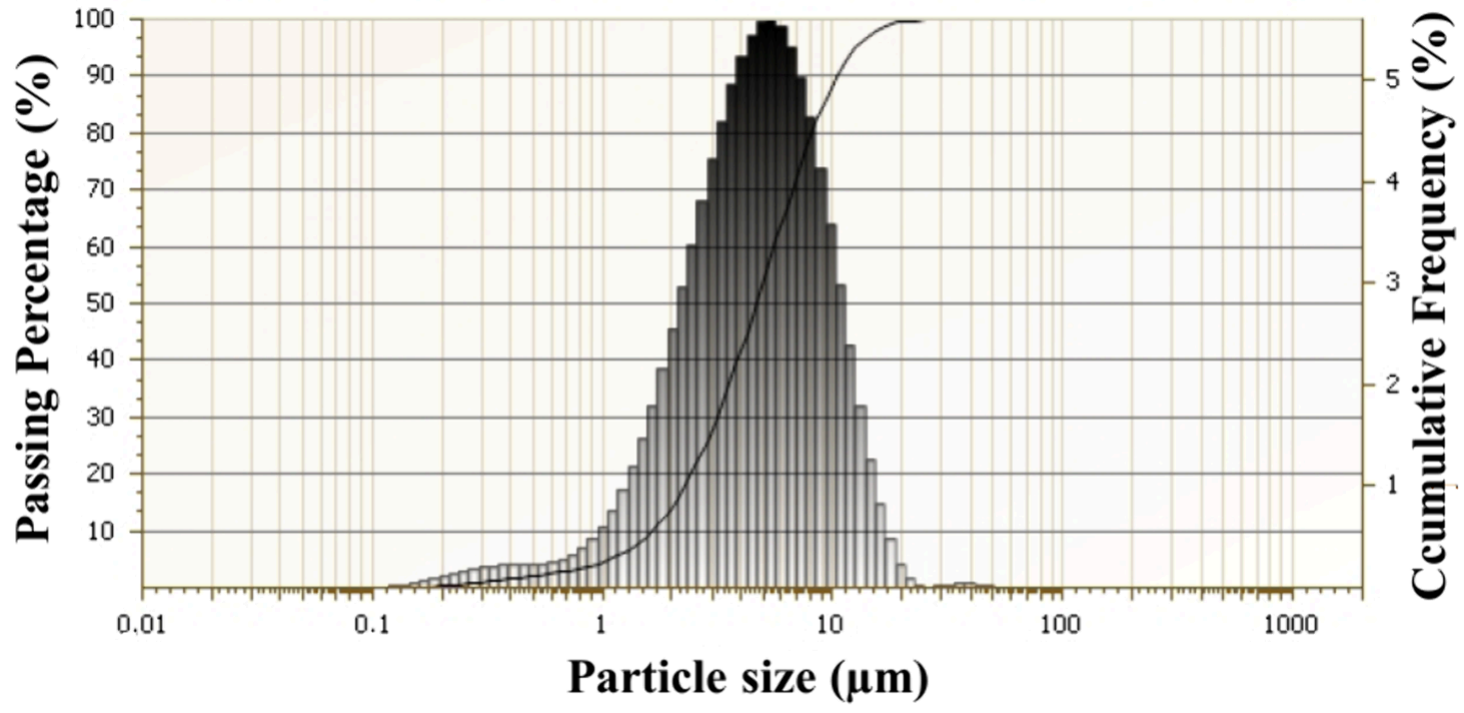
Particle size distribution of metakaolin.

### 3.4 X-ray Diffraction

The X-ray diffraction pattern presented in Figure 5 indicates that the kaolin filler is predominantly composed of kaolinite. The characteristic basal reflection of kaolinite is observed at approximately 12.3° (2θ), together with another intense reflection at about 24.9°, in agreement with PDF card 14–0164. Additionally, a weaker peak near 21°, is attributed to quartz (PDF 05–0490), indicating its presence as a minor phase.

**Figure 5:**
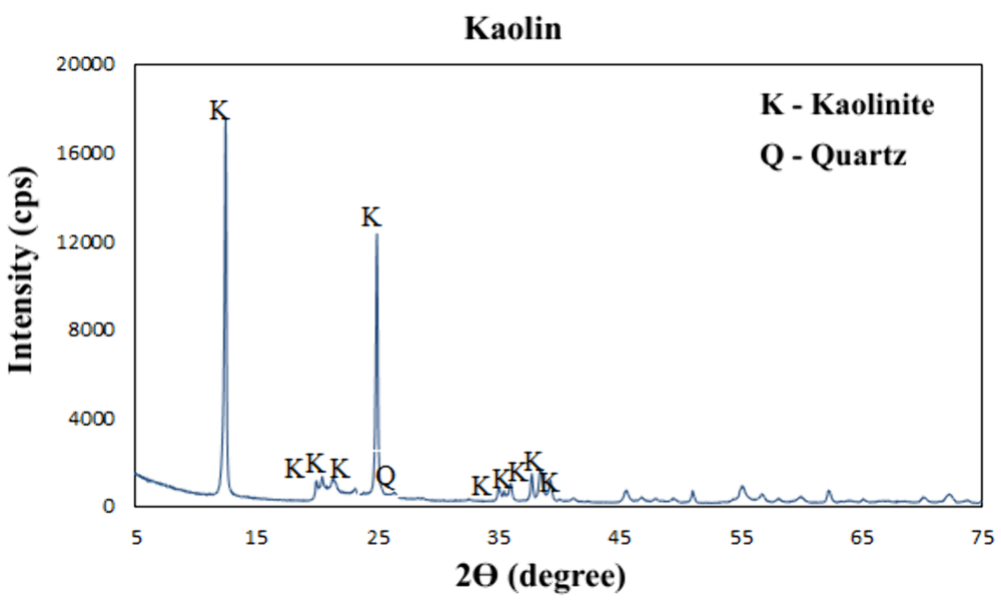
XRD pattern of the kaolin filler.

Figure 6 shows the diffractogram of the calcined material, whose result indicates that it is an amorphous material, as is characteristic of metakaolinite, with the presence of only one quartz peak.

**Figure 6:**
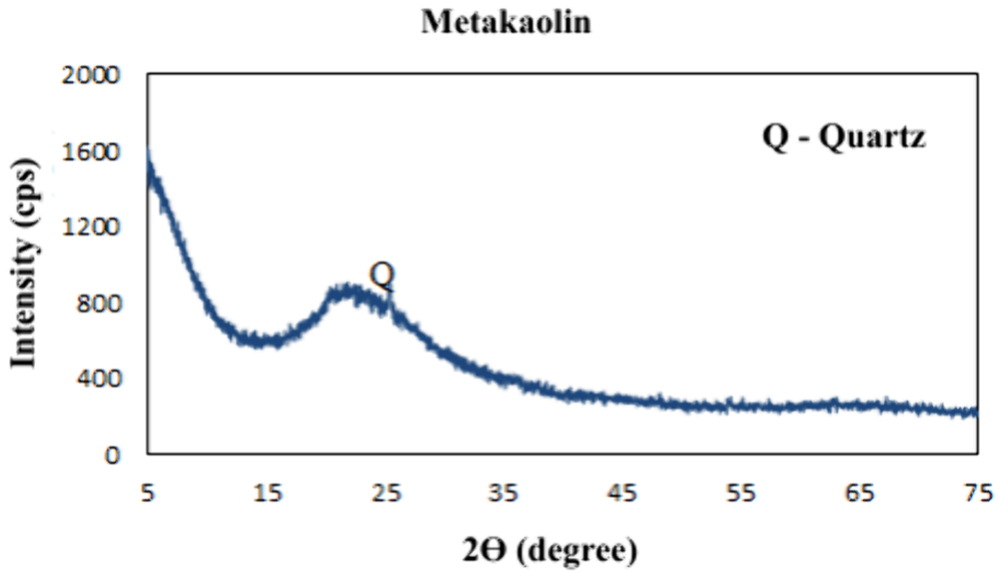
XRD pattern of the metakaolin.

The metakaolin obtained after calcination was used as the precursor for zeolite synthesis. The effects of reaction time and Si/Al ratio on the product were systematically investigated, as summarized in Table 3. Additional results obtained under varying experimental conditions are provided in the Supporting Information.

**Table 3:** Synthesis Conditions of the Zeolitic Product at Different Experimental Times^a^

PZ-X-Y-Z	
Experiments	XRD
	PZ-0.57-60-03
	PZ-0.57-120-03
	PZ-0.57-180-03

a PZ: zeolite product; X: Al/Na ratio; Y: time (min); Z: experiment.

Considering the nomenclature PZ-0.57-60-03, presented in Table 3, this refers to the zeolitic product synthesized with an Al/Na ratio of 0.57, synthesis time of 60 min, obtained in the third experiment. The other nomenclatures follow the same standardization, allowing systematic identification of the experimental conditions used.

Figure 7 shows the diffractograms obtained for the experiments conducted at synthesis times of 60, 120, and 180 min, enabling analysis of the influence of reaction time on the formation of crystalline phases in the synthesized material.

**Figure 7:**
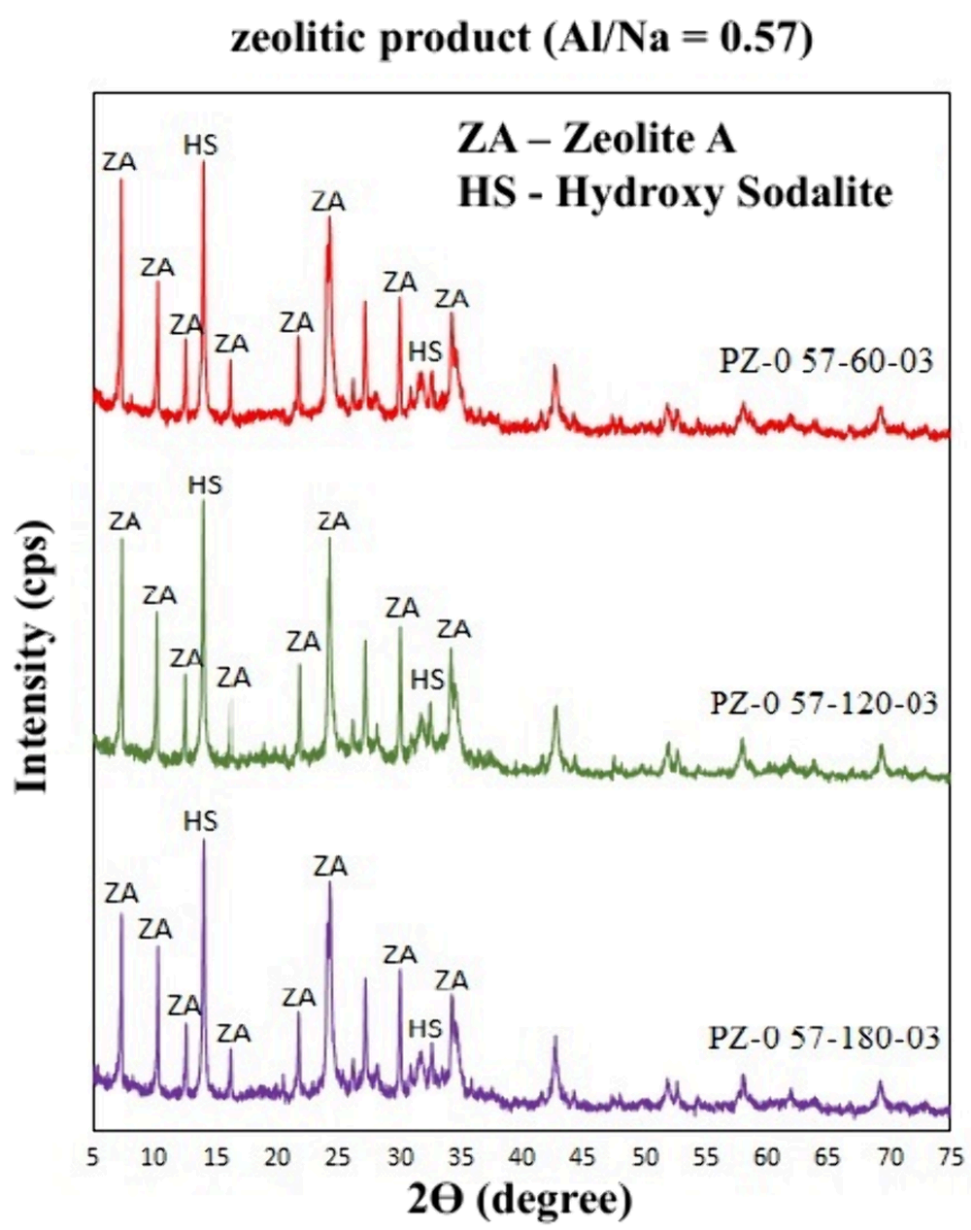
XRD patterns of the zeolitic material synthesized at 60, 120, and 180 min.

Figure 7 shows the main characteristic peaks of NaA zeolite, as identified by PDF standard 089-8015. These results indicate that the synthesis conditions adopted, temperature between 100 and 120 °C, Al/Na ratio = 0.57, pressure of 1.5 Kgf/cm^2^, and maximum time of 180 min, were favorable for the formation of this type of zeolite, allowing the system to reach structural stability.

In addition, hydroxysodalite was observed in all diffractograms, with no significant differences between the synthesis times evaluated. Even for the shortest time tested (30 min; see Supporting Information), the synthesized products showed similar characteristics, suggesting that prolonged synthesis times are not necessary. These findings reinforce that the semipilot scale production system with steam heating has proven effective in obtaining NaA-type zeolite.

Phase identification and confirming the formation of zeolite A under semipilot synthesis conditions were the main goals of the XRD analysis. The diffraction patterns showed the distinctive reflections of zeolite A, indicating successful crystallization. Complementary methods like SEM, XRF, and TG/DTA analyses further supported the structural interpretation by confirming the stability and formation of the synthesized material.

### 3.5 Scanning Electron Microscopy

Figure 8 shows a micrograph of the kaolin filler, revealing a pseudohexagonal flaky morphology, typical of kaolinitic clays, as described by Schwanke et al.^3^

**Figure 8:**
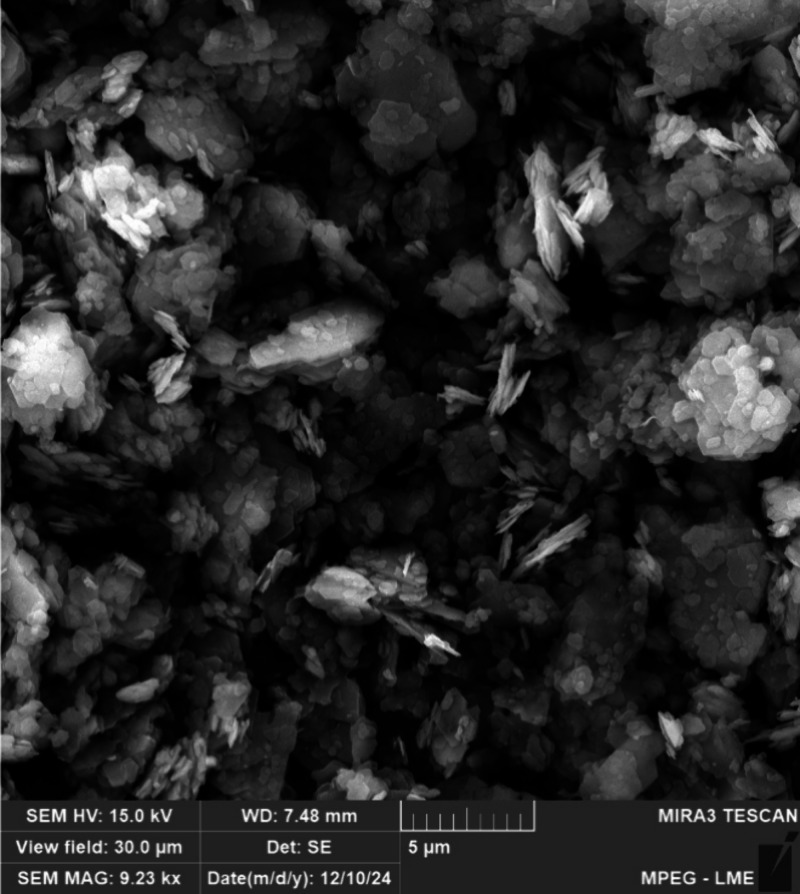
SEM micrographs of the kaolin filler.

Figure 9 shows the scanning electron microscopy of metakaolin, whose micrograph reveals a pseudohexagonal morphology, like the one observed in the analysis of kaolin. In addition, the presence of strongly agglomerated particles is noted, a characteristic that may influence their properties and reactivity in subsequent processes.

**Figure 9:**
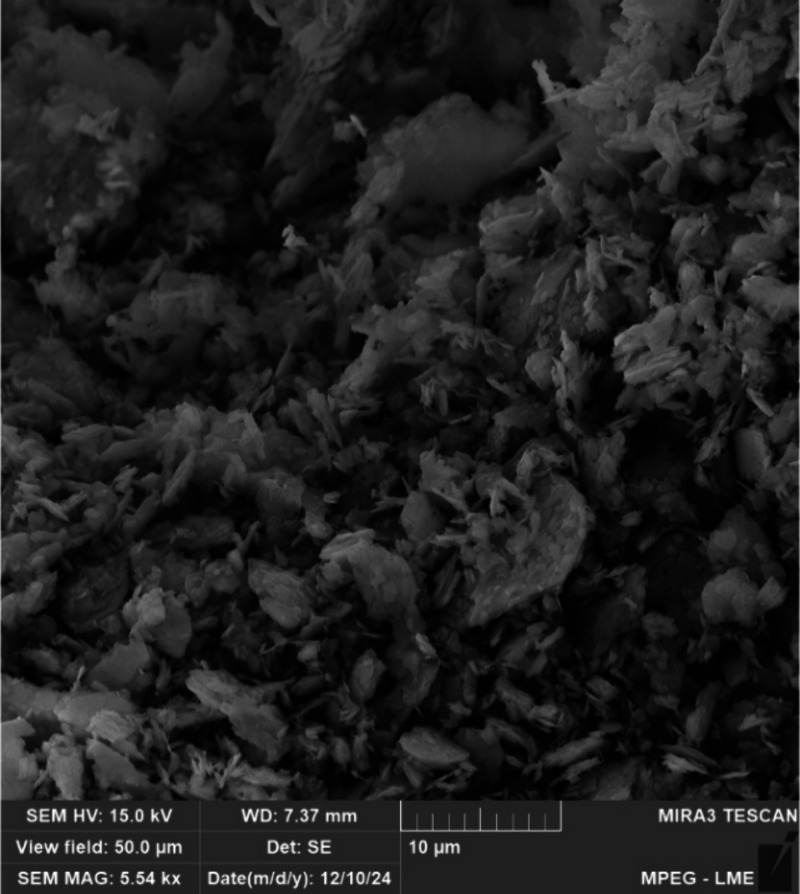
SEM micrographs of the metakaolin.

Scanning electron microscopy analysis was performed for the 180 min experiment, allowing the evaluation of the morphology of the zeolitic product used in the adsorption assays. Figure 10 presents the SEM results obtained for experiment PZ-0.57-180-01, showing cubic and spherical morphologies characteristic of zeolite A and hydroxysodalite, respectively.

**Figure 10:**
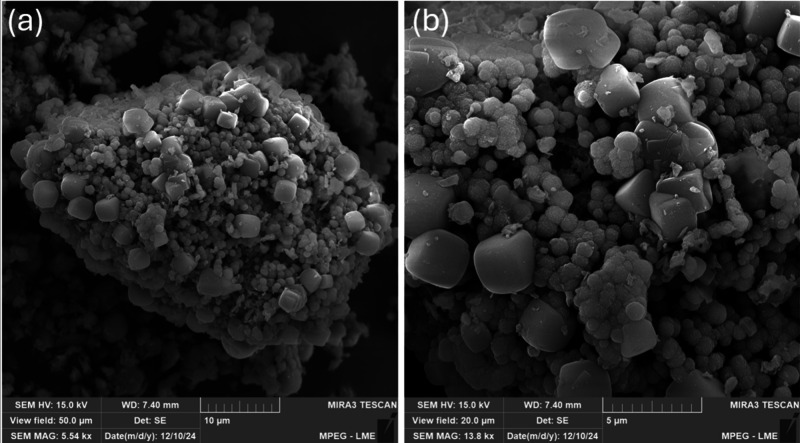
SEM micrographs of zeolite products synthesized at Al/Na = 0.57 and a synthesis time of 180 min, with scale bars of (a) 10 μm and (b) 5 μm.

The results of scanning electron microscopy, shown in Figures 10a-b, revealed a cubic morphology characteristic of zeolite A, in addition to the presence of several spherical crystals, indicating the formation of the sodalite zeolite phase. In Figure 10a, a considerable amount of agglomerated crystals and small portions of unreacted kaolinite were observed, attributed to the shorter synthesis time evaluated. However, this condition did not prevent the formation of the zeolitic product of interest.

### 3.6 Copper Ion Adsorption

The zeolitic product synthesized in this study was subjected to adsorption experiments to evaluate its effectiveness as an adsorbent solid. For this purpose, kinetics, equilibrium, pH variation, and adsorbent mass tests were performed.

### 3.7 Adsorption Kinetics, pH Variation, and Adsorbent Mass

Figure 11 shows the adsorption kinetics of Cu^2+^ ions on the synthesized adsorbent, indicating that adsorption increases over time and reaches equilibrium at approximately 120 min for all concentrations evaluated.

**Figure 11:**
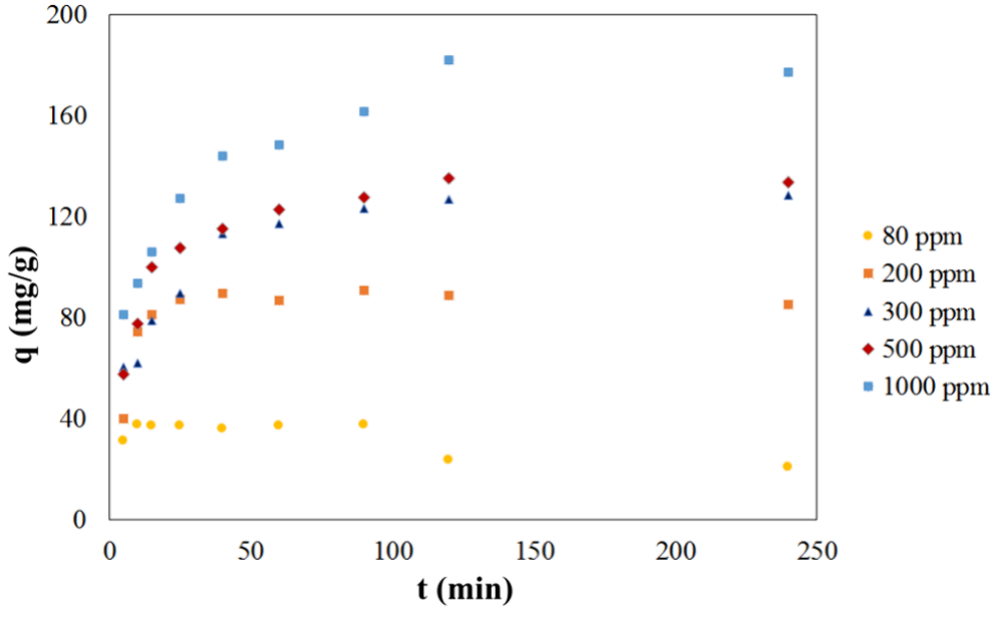
Adsorption kinetics of Cu^2+^ ions.

Previous studies have reported different equilibrium times depending on the zeolite structure and the adsorbate involved. For example, Arslan et al.,^49^ observed equilibrium times of approximately 30 min for NH_4_^+^ adsorption using faujasite zeolites, while Jamil et al.,^50^ reported efficient removal of several metal ions using zeolite A.

The differences in equilibrium time observed among studies may be associated with variations in zeolite framework type, pore structure, and physicochemical properties of the adsorbate. In the present work, the kinetic profile suggests a gradual occupation of available active sites, with equilibrium being achieved after approximately 120 min.

Figure 12 shows the adsorption capacity of the synthesized zeolite as a function of pH, considering concentrations of 60 and 500 ppm in three pH ranges: 3, 4, and 5. The results indicate that higher pH values favor adsorption. A similar trend was reported by Chen et al.,^51^ who synthesized zeolite 4A and attributed the increase in adsorption capacity at higher pH to less competition for active sites between hydrogen ions and Sr­(II) metal. At lower pH values, this competition intensified, reducing the adsorption of Sr­(II).

**Figure 12:**
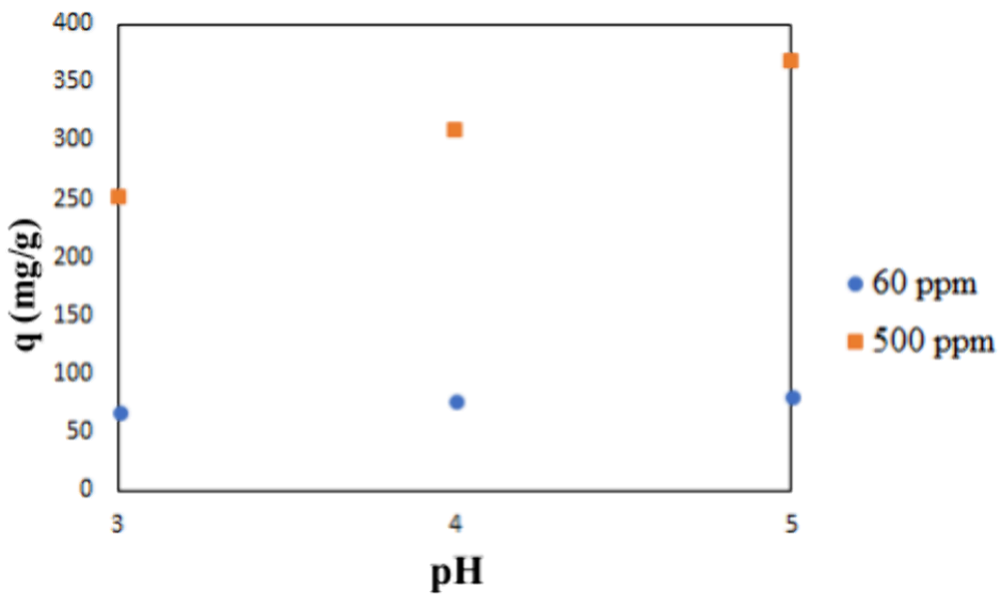
Effect of pH on the adsorption capacity of Cu^2+^ at different initial concentrations.

The influence of the variation in adsorbent mass on the adsorption experiments was evaluated and is shown in Figure 13. The analysis was conducted for concentrations of 80, 300, and 1000 ppm. The results indicated that increasing the amount of adsorbent reduced the adsorption capacity, suggesting that a large amount of material is not necessary for the efficient removal of copper ions (Cu^2+^).

**Figure 13:**
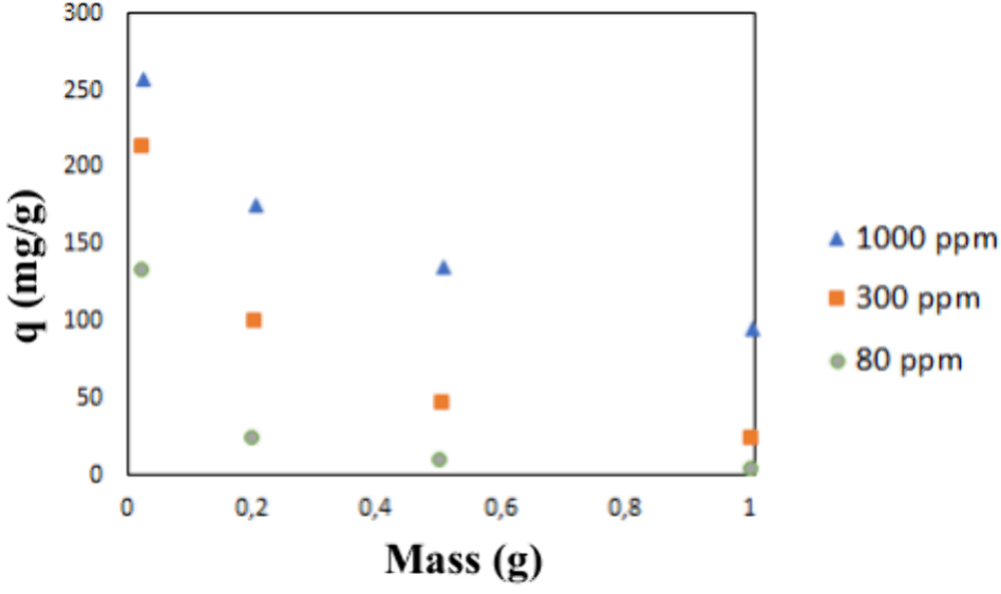
Effect of variation in adsorbent mass on the adsorption capacity (q) of Cu^2+^ at different initial concentrations (80, 300, and 1000 ppm).

### 3.8 Adsorption Equilibrium and Estimation of Isothermal Parameters

The equilibrium data were obtained at four different temperatures: 25 °C, 50 °C, and 75 °C. Figure 14a-c shows the resulting equilibrium curves, highlighting the effectiveness of the synthesized zeolite in the adsorption of copper ions. It also shows the results of the estimates obtained with MCMC for the Langmuir, Freundlich, and Sips isotherms.

**Figure 14:**
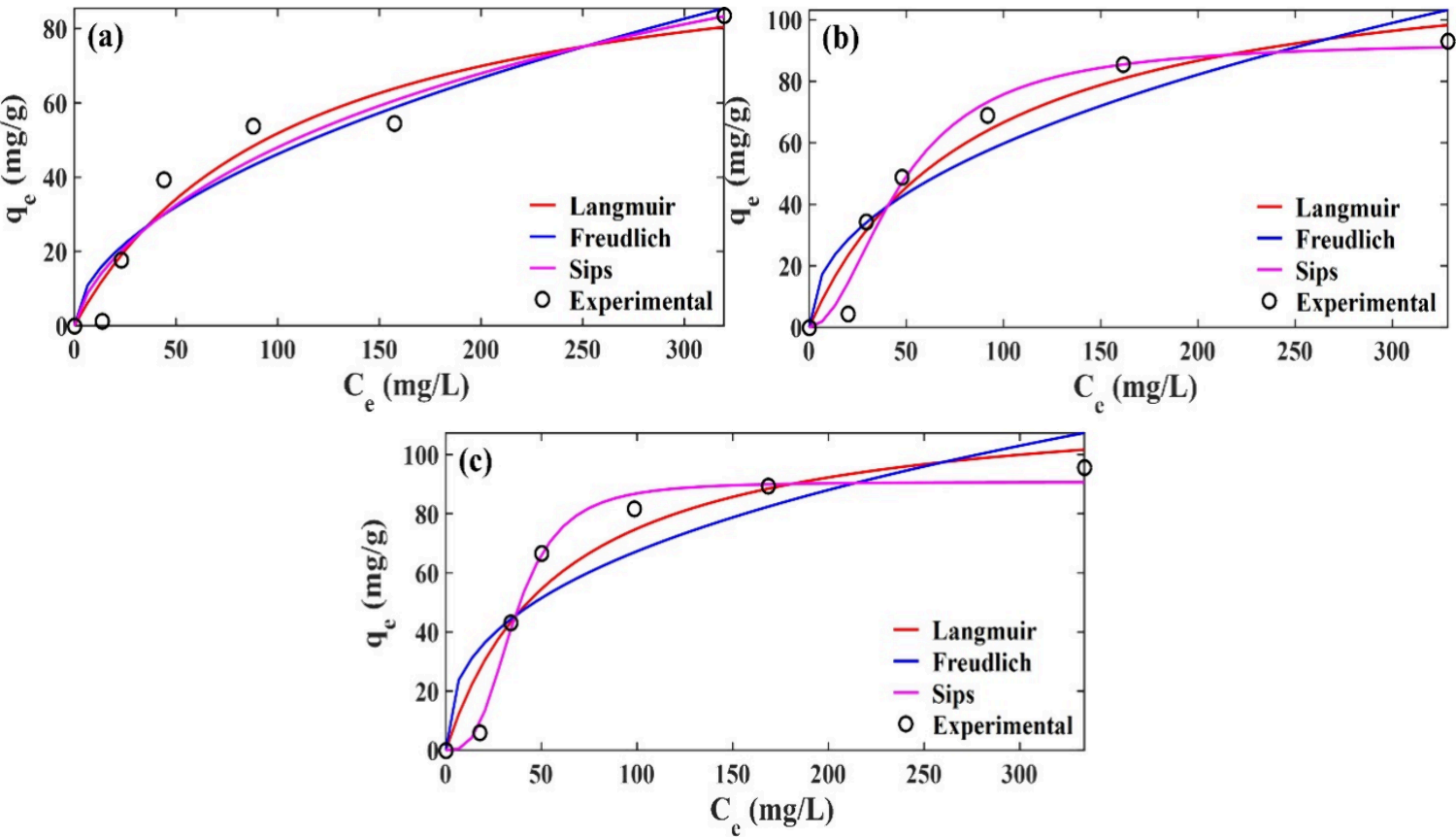
Adsorption isotherms of Cu^2+^ onto the synthesized zeolite at (a) 25 °C, (b) 50 °C, and (c) 75 °C.

The equilibrium curves shown in Figure 14a-c exhibited an initial increase in q_e_ at low equilibrium concentrations. This behavior is associated with the high availability of active adsorption sites on the surface of zeolite. As the Cu^2+^ concentration increases, progressive site occupation leads to a reduction in the slope of the curves, indicating the approach to saturation.

A temperature of 75 °C exhibited the highest adsorption capacity, demonstrating the most efficient performance in experimental terms. However, considering practical applicability in aqueous systems and the cost–benefit balance, a temperature of 50 °C also provided satisfactory results, representing a more feasible and economical option for real processes with lower thermal energy consumption. At lower temperatures, such as 25 °C, the amount of adsorbed Cu^2+^ ions was significantly lower compared to both 50 and 75 °C conditions.

The ideal temperature for achieving good adsorption results was 75 °C. In comparison to the other evaluated conditions, the amount of adsorbed Cu^2+^ ions was significantly lower at lower temperatures, such as 25 °C. As a result, 75 °C is clearly the most effective temperature for the adsorption tests, offering improved performance while preserving advantageous operating conditions and energy consumption. The MCMC method proved to be an effective approach for estimating the parameters of the adsorption isotherms, showing that the Langmuir, and Sips models were in good agreement with the experimental data at temperatures of 25, 50, and 75 °C. Based on the Bayesian information criterion presented in Table 4, it was possible to quantitatively identify the most appropriate isotherm to represent the experimental conditions under which the data were obtained. Table 4 presents the values obtained for the Bayesian model selection metric, where the lowest value recorded indicates the model most likely to adequately represent the physical phenomenon under study.

**Table 4:** Isotherm Models with Obtained BIC Values

	Temperature (°C)		
Isotherm	25	50	75
Langmuir	**36.15**	37.76	40.25
Freundlich	38.20	43.71	48.47
Sips	37.13	**34.61**	**33.30**

At 25 °C, the Langmuir model presented the lowest Bayesian information criterion (36.15), although the Sips isotherm showed a very similar value (37.13). In contrast, at 50 and 75 °C the Sips isotherm produced the lowest BIC values, demonstrating a superior description of the adsorption equilibrium at elevated temperatures. The Freundlich model exhibited higher BIC values at all temperatures and was therefore less adequate for representing the experimental data.

Table 5 presents the estimated values for the parameters obtained using the MCMC methodology, which can be used as a priori information in future investigations involving Bayesian modeling and adsorption phenomena. In addition, R^2^ values are reported to support the evaluation of model performance. The results indicate that the Langmuir model provides the best fit at 25 °C, whereas the Sips isotherm performs better at 50 and 75 °C.

**Table 5:** Estimated values for parameters with MCMC

Model		Temperature (°C)		
Isotherms	Parameters	25	50	75
Langmuir	K (L/mg)	0.01	0.01	0.02
	q_m_ (mg/g)	107.52	124.10	119.89
	R^2^	0.95	0.94	0.94
Freundlich	K_F_ (mg/g)(L/mg)^1/n^	4.04	7.22	11.33
	n (−)	1.89	2.18	2.58
	R^2^	0.92	0.88	0.84
Sips	K_*S*_ (L/mg)	195.29	93.09	90.75
	β_*S*_ (−)	0.71	1.97	3.07
	*a*_S_ (L/mg)	0.002	0.02	0.03
	R^2^	0.93	0.98	0.99

## 4 Conclusion

Kaolin filler was successfully used as a precursor in the hydrothermal synthesis of zeolitic materials. The heat required for the reaction was provided by steam generated in a boiler, allowing precise control of operating variables such as system temperature and pressure and significantly reducing the synthesis time to just 3 h. The method and the scale employed in this study represent a viable alternative to producing zeolites in larger quantities than those typically obtained in laboratory-scale experiments.

The adsorption data were best described by the Langmuir model at 25 °C and by the Sips isotherm at 50 and 75 °C, as indicated by both R^2^ and the Bayesian Information Criterion. Parameters estimated using the MCMC method provide a reliable basis for future investigations involving Bayesian modeling and can serve as prior information for subsequent studies.

The obtained zeolitic product proved effective for the adsorption of copper ions, a potentially toxic element, exhibiting high removal capacity under the tested conditions. Its performance highlights the material’s suitability for treating industrial or highly concentrated effluents and demonstrates the practical potential of the synthesized zeolite for environmental remediation applications.

## Supplementary Material



## Notes

Additional information on the synthesis and characterization of zeolitic products, including the synthesis conditions evaluated at different reaction times (Table S1); calibration curve used for Cu^2+^ quantification (Figure S1); X-ray diffraction patterns of the materials obtained after 30, 60, 120, and 180 min of synthesis (Figures S2–S5); and scanning electron microscopy micrographs illustrating the morphology of selected zeolite samples (Figures S6–S8) (PDF)

## References

[1] Agência Nacional de Mineração (ANM). Anuário Mineral Brasileiro Interativo. https://app.powerbi.com/view?r=eyJrIjoiODIyOWJlMTgtZTBlNi00ODFhLWJiOGEtYzlmOWM3MjhmMWQ4IiwidCI6ImEzMDgzZTIxLTc0OWItNDUzNC05YWZhLTU0Y2MzMTg4OTdiOCJ9.

[2] Murray, H. H. Chapter 5 Kaolin Applications; Applied Clay Mineralogy - Occurrences, Processing and Application of Kaolins, Bentonites, Palygorskite-Sepiolite, and Common Clays 2006; pp 85–109. 10.1016/S1572-4352(06)02005-8.

[3] Schwanke A. J., Silveira D. R., Saorin
Puton B. M., Cansian R. L., Bernardo-Gusmão K. (2022). Sustainable
Conversion of Brazilian Amazon Kaolin Mining Waste to Zinc-Based Linde
Type A Zeolites with Antibacterial Activity. J. Clean Prod.

[4] da
Trindade L. G., Assis M., Souza J. C., Trench A. B., la Rosa Y. N., Teodoro M. D., Schwanke A. J., Longo E., Perrechil F., Braga A. R. C. (2024). Improving the Photocatalytic Dye
Degradation Performance and Bactericidal Properties of Brazilian Amazon
Kaolin-Waste by Adding ZnO and Ag3PO4. J. Alloys
Compd..

[5] Gandhi D., Bandyopadhyay R., Soni B. (2021). Zeolite Y from Kaolin Clay of Kachchh,
India: Synthesis, Characterization and Catalytic Application. Journal of the Indian Chemical Society.

[6] Collins F., Rozhkovskaya A., Outram J. G., Millar G. J. (2020). A Critical Review
of Waste Resources, Synthesis, and Applications for Zeolite LTA. Microporous Mesoporous Mater..

[7] Bulut B., İşbilen E., Atakül H., Tantekin-Ersolmaz Ş.
B. (2022). Adsorptive Removal of
Dimethyl Disulfide
and Thiophene from Liquefied Petroleum Gas by Zeolite-Based Adsorbents. Microporous Mesoporous Mater..

[8] Al-dahri T., AbdulRazak A. A., Rohani S. (2022). Preparation and Characterization
of Linde-Type A Zeolite (LTA) from Coal Fly Ash by Microwave-Assisted
Synthesis Method: Its Application as Adsorbent for Removal of Anionic
Dyes. International Journal of Coal Preparation
and Utilization.

[9] Boer D. G., Langerak J., Bakker B., Pescarmona P. P. (2022). Binderless
Zeolite LTA Beads with Hierarchical Porosity for Selective CO2 Adsorption
in Biogas Upgrading. Microporous Mesoporous
Mater..

[10] Guaya D., Cobos H., Camacho J., López C. M., Valderrama C., Cortina J. L. (2022). LTA and FAU-X Iron-Enriched
Zeolites:
Use for Phosphate Removal from Aqueous Medium. Materials.

[11] Cundy C. S., Cox P. A. (2003). The Hydrothermal
Synthesis of Zeolites: History and
Development from the Earliest Days to the Present Time. Chem. Rev..

[12] Li G., Li M., Zhang X., Cao P., Jiang H., Luo J., Jiang T. (2022). Hydrothermal Synthesis of Zeolites-Calcium Silicate Hydrate Composite
from Coal Fly Ash with Co-Activation of Ca­(OH)­2-NaOH for Aqueous Heavy
Metals Removal. Int. J. Min Sci. Technol..

[13] Xie W.-M., Zhou F.-P., Bi X.-L., Chen D.-D., Li J., Sun S.-Y., Liu J.-Y., Chen X.-Q. (2018). Accelerated Crystallization
of Magnetic 4A-Zeolite Synthesized from Red Mud for Application in
Removal of Mixed Heavy Metal Ions. J. Hazard
Mater..

[14] Jin Y., Li L., Liu Z., Zhu S., Wang D. (2021). Synthesis and Characterization
of Low-Cost Zeolite NaA from Coal Gangue by Hydrothermal Method. Advanced Powder Technology.

[15] Sun X., Wang J., Jiang Y., Maturura E., Wang W., Yang R., Xing C., Chen J., Tsubaki N. (2022). Facile Synthesis
of Zeolites under an Atmospheric Reflux System. Microporous Mesoporous Mater..

[16] Sazali N., Harun Z. (2022). One Shot of the Hydrothermal Route for the Synthesis of Zeolite LTA
Using Kaolin. J. Inorg. Organomet Polym. Mater..

[17] Brindley G. W., Nakahira M. (1959). The Kaolinite-Mullite Reaction Series: II, Metakaolin. J. Am. Ceram. Soc..

[18] Chen X., Tao J., Sun P., Yu F., Li B., Dun L. (2022). Effect of
Calcination on the Adsorption of Chifeng Zeolite on Pb2+ and Cu2+. International Journal of Low-Carbon Technologies.

[19] Radhika M., Palanivelu K. (2006). Adsorptive
Removal of Chlorophenols from Aqueous Solution
by Low Cost Adsorbent—Kinetics and Isotherm Analysis. J. Hazard Mater..

[20] McCabe, W. L. ; Smith, J. C. ; Harriot, P. Adsorption. In Unit Operations of Chemical Engineering; McGraw Hill: New York, 1993; pp 810–837.

[21] Dąbrowski A. (2001). Adsorption
— from Theory to Practice. Adv. Colloid
Interface Sci..

[22] Günay A., Arslankaya E., Tosun İ. (2007). Lead Removal from Aqueous Solution
by Natural and Pretreated Clinoptilolite: Adsorption Equilibrium and
Kinetics. J. Hazard Mater..

[23] Aharoni C., Ungarish M. (1977). Kinetics of Activated
Chemisorption. Part 2.—Theoretical
Models. Journal of the Chemical Society, Faraday
Transactions 1: Physical Chemistry in Condensed Phases.

[24] Ho Y. (2006). Review of
Second-Order Models for Adsorption Systems. J. Hazard Mater..

[25] Nagy B., Mânzatu C., Măicăneanu A., Indolean C., Barbu-Tudoran L., Majdik C. (2017). Linear and Nonlinear
Regression Analysis
for Heavy Metals Removal Using Agaricus Bisporus Macrofungus. Arabian Journal of Chemistry.

[26] Royer B., Cardoso N. F., Lima E. C., Vaghetti J. C. P., Simon N. M., Calvete T., Veses R. C. (2009). Applications
of Brazilian Pine-Fruit
Shell in Natural and Carbonized Forms as Adsorbents to Removal of
Methylene Blue from Aqueous Solutions—Kinetic and Equilibrium
Study. J. Hazard Mater..

[27] Moraes N. L., de Vilhena M. B., Rossi D. M., Viegas B. M. (2025). Mathematical Modeling
and Simulation of 1,3-Propanediol Production by *Klebsiella
Pneumoniae* BLh-1 in a Batch Bioreactor Using Bayesian Statistics. Biotechnol. Bioeng..

[28] Lima H. B. S., Sousa A. P. S. de, Silva W. B. da, Costa D. S. da, Rodrigues E. C., Estumano D. C. (2024). Parameter Estimation of Breakthrough
Curve Models in the Adsorption Process of H2S and CO2 Using the Markov
Chain Monte Carlo Method. Applied Sciences.

[29] Soeiro W. F., Moura C. H. R., Dias C. S., Rodrigues E. C., Da Costa D. S., Viegas B. M., Estumano D. C. (2024). Mathematical
Evaluation
of Direct and Inverse Problem Applied in Breakthrough Models of Metal
Adsorption. Applied Sciences.

[30] Cardoso A. C., Dias C. S., Moura C. H. R. de, Ferreira J. L., Rodrigues E. C., Macêdo E. N., Estumano D. C., Viegas B. M. (2024). Use of Bayesian
Methods in the Process of Uranium Bioleaching by Acidithiobacillus
Ferrooxidans. Applied Sciences.

[31] Tavares R. S., Dias C. S., Moura C. H. R., Rodrigues E. C., Viegas B. M., Macêdo E. N., Estumano D. C. (2022). Parameter Estimation
in Mass Balance Model Applied in Fixed Bed Adsorption Using the Markov
Chain Monte Carlo Method. Journal of Heat and
Mass Transfer Research.

[32] Moura C. H. R., Viegas B. M., Tavares M. R. M., Macêdo E. N., Estumano D. C., Quaresma J. N. N. (2021). Parameter
Estimation in Population
Balance Through Bayesian Technique Markov Chain Monte Carlo. Journal of Applied and Computational Mechanics.

[33] Amador I. C. B., Nunes K. G. P., de
Franco M. A. E., Viegas B. M., Macêdo E. N., Féris L. A., Estumano D. C. (2022). Application of Approximate Bayesian
Computational Technique to Characterize the Breakthrough of Paracetamol
Adsorption in Fixed Bed Column. International
Communications in Heat and Mass Transfer.

[34] de
Moura C. H. R., Viegas B. M., Madruga Tavares M. R., Macêdo E. N., Estumano D. C. (2022). Estimation of Parameters and Selection
of Models Applied to Population Balance Dynamics Via Approximate Bayesian
Computational. Journal of Heat and Mass Transfer
Research.

[35] Viegas B. M., Magalhães E. M., Orlande H. R. B., Estumano D. C., Macêdo E. N. (2023). Experimental
Study and Mathematical Modelling of Red Mud Leaching: Application
of Bayesian Techniques. International Journal
of Environmental Science and Technology.

[36] Dias C. S., Franco M. A. E., Rodrigues E. C., Ferreira J. L., Viegas B. M., Féris L. A., Estumano D. C., Macêdo E. N. (2024). Diclofenac
Sodium Adsorption on Activated Carbon: Experimental, Modeling and
Bayesian Statistics. An Acad. Bras Cienc.

[37] Formigosa L. F., dos Santos I. C., Alves e Álvares L. E., Macêdo E. N., Gonçalves L. R.
B., Viegas B. M. (2026). Mathematical
Modeling
of Amoxicillin Synthesis in Batch and Semi-Batch Reactor: Application
of Bayesian Statistics and Genetic Algorithm. Biotechnol. Bioeng..

[38] Martins
Neto A. P., Tavares A. L. S., Formigosa L. F., Gomes B. D., Gonçalves L. R.
B., Viegas B. M. (2025). Enzymatic
Synthesis of Amoxicillin in a Batch Reactor: Mathematical Modeling,
Sensitivity Analysis, and Experimental Validation. ACS Omega.

[39] de
Moura C. H. R., da Silva C. A. M., Ferreira J. L., Macêdo E. N., Quaresma J. N. N., Cotta R. M. (2025). Analysis of Binding Kinetics and
Mass Transport in SPR-Based Biosensor Using the Generalized Integral
Transform Technique and the Markov Chain Monte Carlo Method. Comput. Biol. Med..

[40] Zhou J., Liu H., Liu D., Yuan P., Bu H., Du P., Fan W., Li M. (2022). Sorption/Desorption of Eu­(III) on Halloysite and Kaolinite. Appl. Clay Sci..

[41] Mahima
Kumar M., Irshad K. A., Jena H. (2021). Removal of Cs+ and
Sr2+ Ions from Simulated Radioactive Waste Solutions Using Zeolite-A
Synthesized from Kaolin and Their Structural Stability at High Pressures. Microporous Mesoporous Mater..

[42] Abubakar M., Muthuraja A., Ahmad N. (2021). Experimental Investigation of the
Effect of Temperature on the Density of Kaolin Clay. Mater. Today Proc..

[43] Tada S., Li D., Okazaki M., Kinoshita H., Nishijima M., Yamauchi N., Kobayashi Y., Iyoki K. (2023). Influence of Si/Al
Ratio of MOR Type Zeolites for Bifunctional Catalysts Specific to
the One-Pass Synthesis of Lower Olefins via CO2 Hydrogenation. Catal. Today.

[44] Liang D., Liu Y., Zhang R., Xie Q., Zhang L. (2024). A Review on the Influence
Factors in the Synthesis of Zeolites and the Transformation Behavior
of Silicon and Aluminum During the Process. Comments on Inorganic Chemistry.

[45] Tian Q., Yu X., Sui Y., Lina X., Lv Z. (2022). The Effect of Water
on Metakaolin Phosphate-Based Geopolymer Properties. Ceramics - Silikaty.

[46] Cheng Y., Xing J., Bu C., Zhang J., Piao G., Huang Y., Xie H., Wang X. (2019). Dehydroxylation and
Structural Distortion of Kaolinite as a High-Temperature Sorbent in
the Furnace. Minerals.

[47] Santos, P. de S. Ciência e Tecnologia de Argilas, 2nd ed.; Edgard Blücher: São Paulo, 1989; Vol. 1.

[48] Bloodworth, A. J. ; Highley, D. E. ; Mitchell, C. J. Industrial Minerals Laboratory Manual: Kaolin; Nottingham, UK, 1993.

[49] Arslan A., Veli S. (2012). Zeolite 13X for Adsorption of Ammonium Ions from Aqueous Solutions
and Hen Slaughterhouse Wastewaters. J. Taiwan
Inst Chem. Eng..

[50] Jamil T. S., Ibrahim H. S., Abd El-Maksoud I. H., El-Wakeel S. T. (2010). Application
of Zeolite Prepared from Egyptian Kaolin for Removal of Heavy Metals:
I. Optimum Conditions. Desalination.

[51] Chen Z., Li X., Liu H., Xu W., Yu J., Zang Y., Hu G., Hu T., Jiang J., Mao P., Pan Y., Wei Y. (2024). A Novel and Cost-Effective Synthesis of Magnetic Zeolite 4A Using
Kaolinite and Red Mud for Sr­(II) Removal. Microporous
Mesoporous Mater..

