# Supplementary material for: Semipilot Scale Synthesis of Zeolite A from Kaolin Filler: Process Optimization with Boiler Steam, Mathematical Modeling, and Cu2+ Adsorption
Source: ACS Omega. 2026 May 28;11(22):32345–55. doi: 10.1021/acsomega.5c13534 (PMC13261423; doi:10.1021/acsomega.5c13534)
Supplement: Supplementary file 1 [file ao5c13534_si_001.pdf]

Supplementary material for: Semi-pilot scale synthesis of zeolite A from kaolin filler: process optimization with boiler steam, mathematical modeling, and Cu^{2+} adsorption

Emerson Cardoso Rodrigues[1,2], Camila Santana Dias[1], Josiel Lobato Ferreira[3], Bruno Maués Farias[1], Diego Cardoso Estumano[3,4,5], Bruno Marques Viegas[3,5], José Antônio da Silva Souza[1], Emanuel Negrão Macêdo[1,2,5,]*

[1]Engineering of Natural Resources of the Amazon, Federal University of Pará, Belém, PA, 66075-110, Brazil.

[2]Faculty of Chemical Engineering, Federal University of Pará, Belém, PA, 66075-110, Brazil.

[3]Faculty of Biotechnology, Federal University of Pará, Belém, PA, 66075-110, Brazil.

[4]Simulation and Computational Biology Laboratory, High Performance Computing Center, Federal University of Pará, Belém, PA, 66075-110, Brazil.

[5]Graduate Program in Chemical Engineering, Federal University of Pará, Belém, PA, 66075-110, Brazil.

mersone7@yahoo.com.br (Emerson Cardoso Rodrigues), camiladias07@outlook.com.br (Camila Santana Dias), jlobato@ufpa.br (Josiel Lobato Ferreira), brunomauesfarias@gmail.com (Bruno Maués Farias), dcestumano@ufpa.br (Diego Cardoso Estumano), viegasmbruno@gmail.com (Bruno Marques Viegas), jass@ufpa.br (José Antônio da Silva Souza), enegrao@ufpa.br (Emanuel Negrão Macêdo)

* Corresponding author.

Mailing address: Engineering of Natural Resources of the Amazon, Federal University of Pará, Belém, PA, 66075-110, Brazil.

E-mail address: enegrao@ufpa.br (Emanuel Negrão Macêdo).

Table S1. Synthesis conditions of the zeolitic product at different experimental times.

	PZ-X-Y-Z	
	XRD	SEM
Experiments	PZ-0.57-30-01<br>PZ-0.57-60-02<br>PZ-0.57-120-02<br>PZ-0.57-180-02	PZ-0.57-60-02<br>PZ-0.57-120-03<br>PZ-0.57-180-03

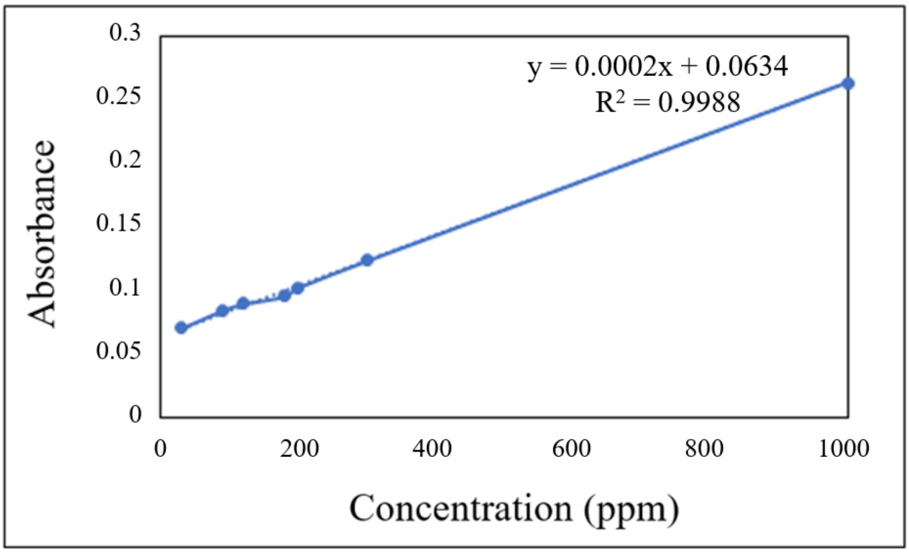

Figure S1. Calibration curve for Cu^{2+} quantification obtained by UV-Vis spectrometry at 800 nm.

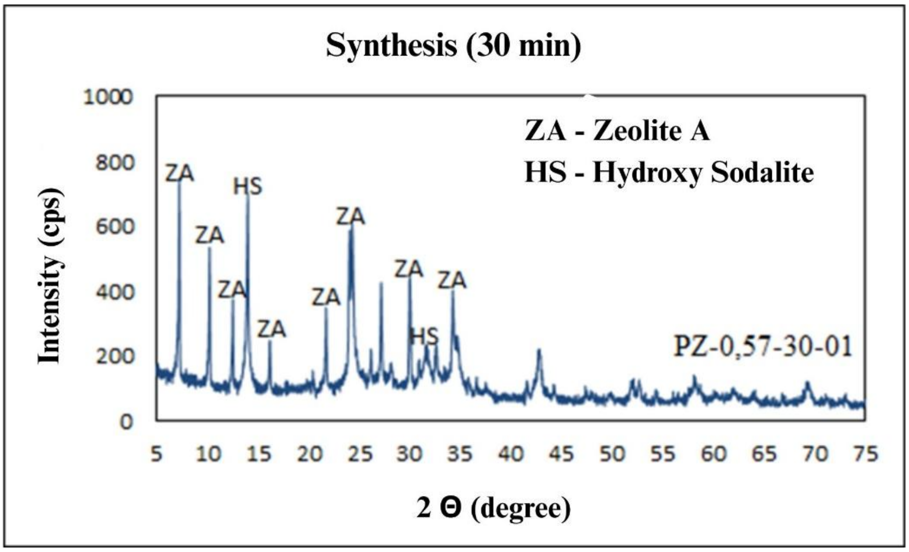

Figure S2. X-ray diffraction patterns of zeolitic products after 30 min of synthesis.

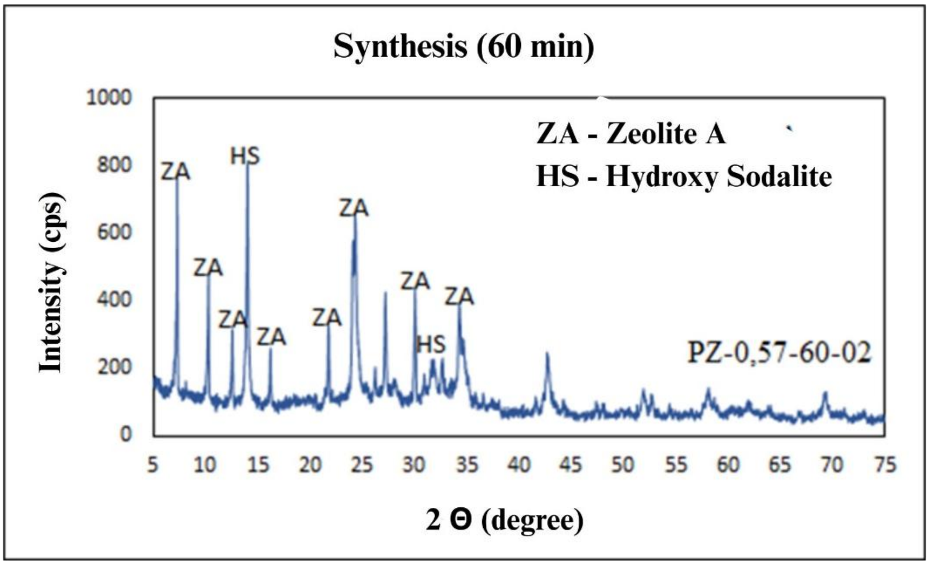

Figure S3. X-ray diffraction patterns of zeolitic products after 60 min of synthesis.

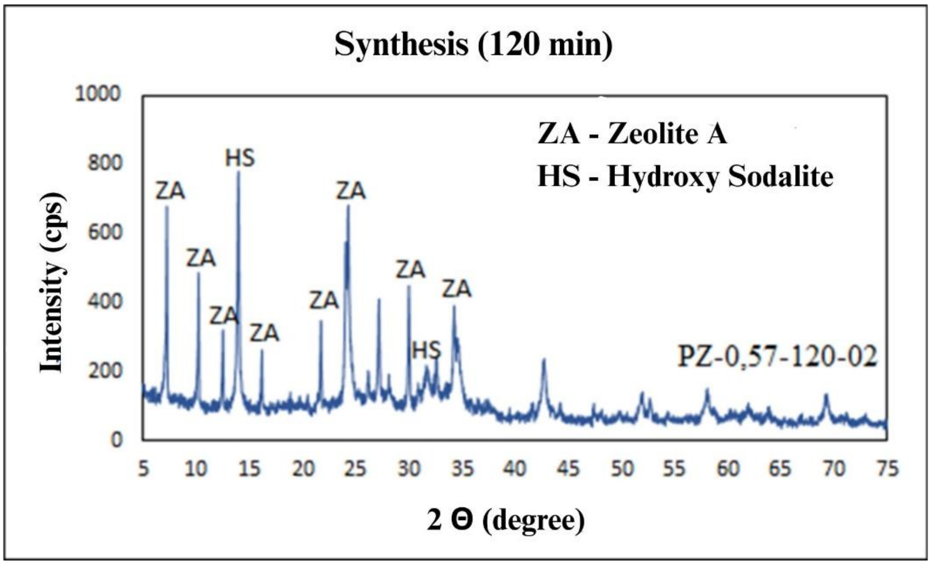

Figure S4. X-ray diffraction patterns of zeolitic products after 120 min of synthesis.

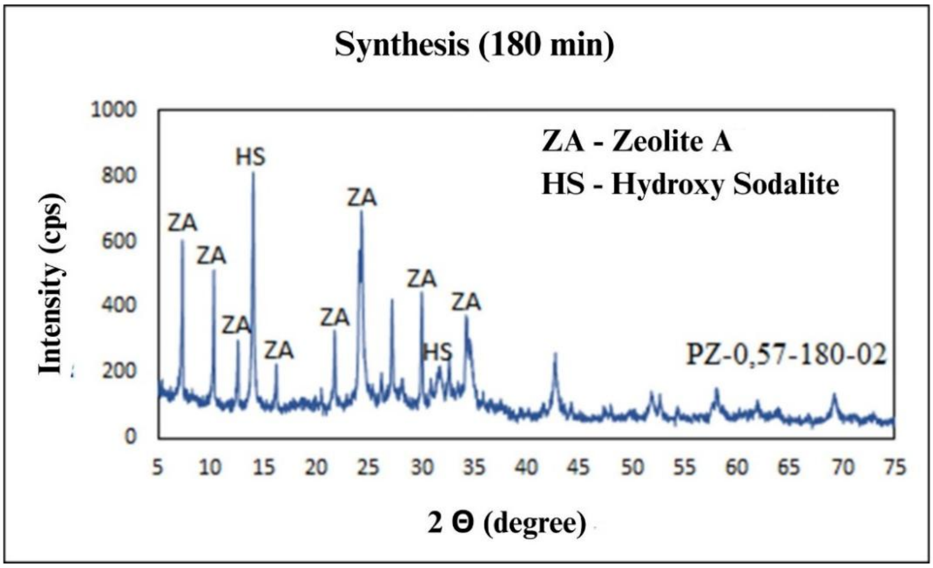

Figure S5. X-ray diffraction patterns of zeolitic products after 180 min of synthesis.

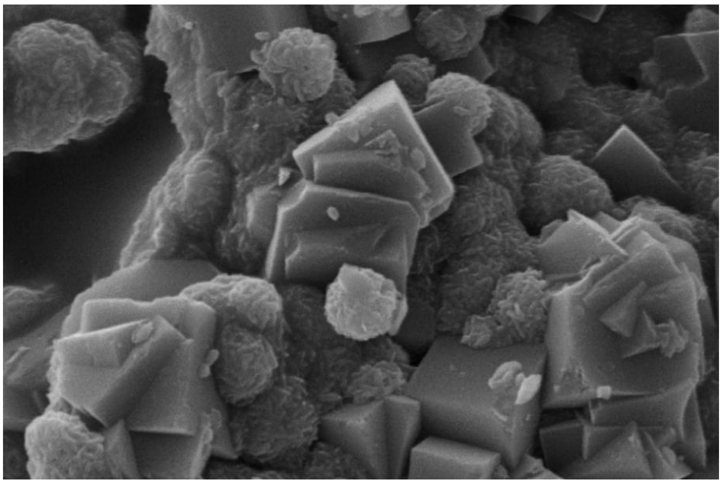

Figure S6. SEM micrographs of the zeolite sample coded PZ-0.57-60-03.

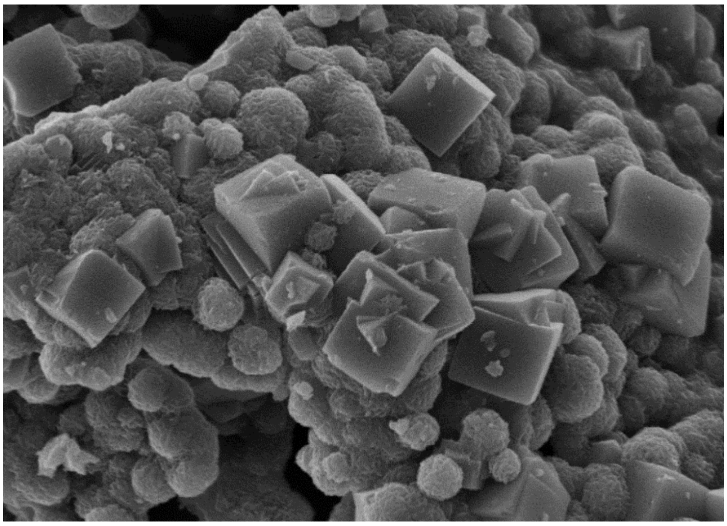

Figure S7. SEM micrographs of the zeolite sample coded PZ-0.57-120-02.

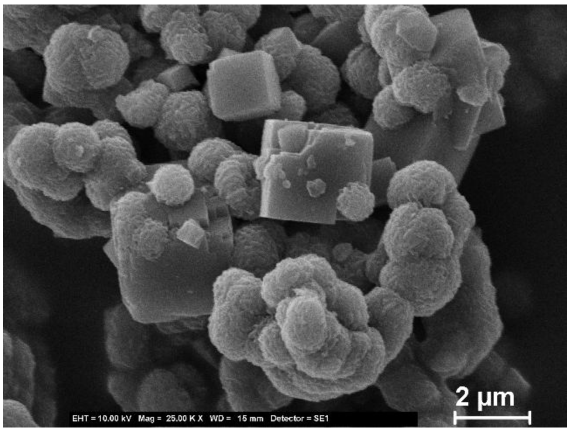

Figure S8. SEM micrographs of the zeolite sample coded PZ-0.57-180-03.
